# The History of the Panmictic Population Concept and Its Legacy in Contemporary Population Genetics

**DOI:** 10.1111/ahg.70015

**Published:** 2025-07-28

**Authors:** Andy Walton, Alex Aylward, Mark G. Thomas, Adam Rutherford

**Affiliations:** ^1^ UCL Genetics Institute, Genetics, Evolution and Environment University College London London UK; ^2^ Faculty of History University of Oxford Oxford UK

**Keywords:** continuous space, isolation by distance, modern synthesis, panmictic population, population genetics, population modelling, population thinking

## Abstract

The panmictic population concept is at the heart of population, evolutionary and conservation genetics. However, in nature, true panmictic populations are vanishingly rare. As an idea conceived for modelling evolutionary dynamics, it has been thought that the assumption of panmixia was formalised during the development of the Modern Synthesis. Here, we show that while the idea's longevity is almost certainly due to its mathematical convenience, it became embedded in evolutionary thought much earlier, initially as a way to reconcile long‐standing essentialist ideas with the advent of Darwin's theories. Though the principles of essentialism and reversion have been largely rejected, these ideas persist in shaping assumptions made about populations in contemporary genetics research, including how they are conceptualised and sampled. This legacy has important implications for the interpretation of genomic findings in human evolution, conservation and medicine. From an evaluation of this history and its legacy, we contend that while the panmictic population concept has been, and continues to be useful, with the generation of terabytes of genomic data in the 21st century, its utility is likely to diminish as the need for continuous space models grows.

## 1 Introduction

In evolutionary biology, a population is typically conceptualised as a group of organisms which interbreed and are geographically isolated, such that there is little or no migration into the group (Dobzhansky 1950). This definition is generally paired with the concept of panmixia (Fisher 1930), which stipulates that the individuals in a given population breed with each other randomly with respect to geography, relatedness, physical appearance and sex ratios. By this working definition, panmictic populations are idealised models and have almost certainly never existed in reality. Nevertheless, science is built on models that are necessarily simplified but are also useful in facilitating our understanding of nature (Bokulich 2011; Box 1976; Morrison 2006).

Most observed genetic differentiation in nature increases with geographic distance for organisms in continuous space, a phenomenon characterised by Sewall Wright as ‘isolation by distance’ (Wright 1943). There are other factors which restrict random mating, such as other geographic barriers to gene flow, assortative mating, inbreeding, imbalanced sex ratios and overlapping generations (Crow and Kimura 1970). With the advent of large‐throughput whole‐genome sequencing, these effects have been demonstrated in a wide array of taxa from local to global scales (David 2018; Manica et al. 2007; Meirmans 2012; Prugnolle et al. 2005; Rosenberg et al. 2005; Serre and Pääbo 2004; Vekemans and Hardy 2004; Wakeley 1999). Despite this, the panmictic population concept continues to be used widely in population, conservation and evolutionary genetics (Allendorf et al. 2013; Battey et al. 2020; Sætre and Ravinet 2019, Chapters 7 and 9).

This poses the two central questions which motivate this present discussion. First, how did the panmictic population concept originate and become embedded in evolutionary and population genetics? And second, why and with what consequences has it persisted in various biological fields?

On the first question, a natural place to begin is with the origins of the biological ‘population’ concept more generally. According to the evolutionary biologist Ernst Mayr, this is one of the many things we owe to Charles Darwin. Mayr urged that, alongside his successes in amassing incontrovertible evidence for evolution, and forwarding a workable and well‐supported evolutionary mechanism, we recognise Darwin's third major achievement, namely that he ‘introduced into the scientific literature a new way of thinking; “population thinking”’ (Mayr 1976, 27). Until the Darwinian era, according to Mayr, philosophers, naturalists and scientists, from Aristotle to Linnaeus, tended to characterise organisms as possessing discrete ‘essences’ and belonging to distinct ‘types’. Only with the 19th‐century recognition of the mutability of species would the grip of essentialism on biology be loosened. By reconceptualising individual variation as a fundamental property of groups of organisms, and the raw material for evolutionary change via natural selection, Darwin thus paved the way for a biology which considered populations seriously.

Though ‘consistently overlooked’ in Mayr's view, this was a move of great significance, particularly given how it set in motion the development of statistical approaches to the study of variation and evolution. It was not Darwin but his half‐cousin, Francis Galton, who would instigate the statistical analysis of organisms (biometry) and thus centre the populational approach. In his 1889 book *Natural Inheritance*, Galton used the term ‘population’ to refer to an abstract statistical grouping from which a sample is derived (Galton 1889; Hey 2011). Despite describing the term as ‘not more than a unit of study’, he also frequently uses ‘population’ to refer to real social or national groupings of people, implying that he viewed populations as both biological entities and statistical groupings (Bertoldi and Pence 2025). Later, following the translation and re‐discovery of Mendel's work, the dominant concept of a population shifted from samples of real individuals to an idealised population of Mendelian alleles, which would allow the implications of Mendelian inheritance in groups to be explored (Fisher 1918).

This oft‐told story takes us from Darwin to the precipice of the Modern Synthesis initiated by the founders of theoretical population genetics, primarily R. A. Fisher, J. B. S. Haldane and Sewall Wright (Provine 2001). By this time, idealised Mendelian populations were (and remain) at the very core of cutting‐edge evolutionary theorising. The question for us is how assumptions about the nature of these populations, specifically the condition that they are panmictic or breed randomly, came to predominate. It is sometimes stated that the assumption of panmixia was incorporated into these idealised population models at the time of the modern synthesis as a necessary mathematical simplification during the model building process (Crow and Kimura 1970, 2 and 32; Haldane 1964; Plutynski et al. 2016; Sterner 2017). In this paper, we re‐evaluate this history and argue that in fact, the notion of panmixia originated earlier and developed in parallel with the growth of population models in evolutionary biology. The fundamental assumption of panmixia was incorporated into evolutionary thought well before the rediscovery of Mendel's work on heredity, and by the time of the Modern Synthesis, it had already become established in the field. The contingent ways in which panmixia became embedded raise questions about its continued centrality in much of population genetic modelling today.

## 2 The Origins of Panmixia

We owe the term panmixia to the German zoologist August Weismann, who coined it in his 1883 essay *On Heredity*. The roots of the idea itself, though, are somewhat older. As is the case with so many central concepts in evolutionary theory, we can find expressions of the panmixia concept in the writings of Darwin.

By the mid‐19th century, the mutability of animals under domestication was broadly accepted (e.g. Buffon et al. 1749; Prichard 1826, 97–98). In *On the Origin of Species*, Darwin was interested in the apparent loss of certain variations among animals under domestication and discussed multiple potential mechanisms to explain this phenomenon. The first sits squarely in the Lamarckian tradition, with Darwin noting that ‘in domestic animals… disuse diminishes [certain parts]; and that such modifications are inherited’ (Darwin 1859, 134). However, later on in *On the Origin of Species*, when discussing ‘rudimentary organs’, Darwin appears to propose an alternative mechanism to Lamarck's for changes under domestication. He states that ‘an organ, when rendered useless, may well be variable, for their variations can no longer be checked by Natural Selection’ (Darwin 1859, 455). He expanded on this in 1868 in the book *The Variation of Animals and Plants Under Domestication*, listing cases of ‘reversion’, stating that ‘unless carefully preserved by man… any particular variation would generally be lost by crossing, reversion, and the accidental destruction of the varying individuals’ (Darwin 1868, 292).

Darwin's idea of reversion by intercrossing was picked up and elaborated on by George Romanes, who stated that the root cause of free intercrossing was the ‘cessation of selection’ (Romanes 1874b). He claimed that in any organ which was no longer affected by selection, the selective pressure would be ‘reversed… through [the] Economy of Growth’. This would cause ‘indiscriminate variation’ in the population, and the cessation of selection would allow less fit individuals ‘a good chance of leaving offspring’ (allowing for intercrossing). However, like Darwin, Romanes still believed that disuse of organs played a part in their reversion (Romanes 1874a).

Here enters August Weismann, who coined the term panmixia as part of his attempt to remove the need for Lamarkian explanations for reversion, arguing that only the absence of selection was necessary. In his 1883 essay *On Heredity*, Weismann states that the ‘degeneration of an organ does not depend on its disuse’. Instead, he writes, the ‘suspension of the preserving influence of natural selection [which] may be termed panmixia’ (Weismann 1883). He later expanded on panmixia's mechanism, stating that ‘as soon as an organ becomes useless… not only those individuals with the best organs have the chance of [reproducing], but also individuals in which the organs are less well developed. Hence follows a mixture of all possible degrees of perfection, which must in the course of time result in the deterioration of the average development of the organ’ (Weismann 1886) and that ‘as soon as natural selection ceases to operate upon any character… it begins to disappear’ (Weismann 1888).

To Weismann, panmixia incorporated several effects. Panmixia occurs when selection is absent, resulting in unrestricted interbreeding (random mating) of all varieties, which thus leads to the reversion of traits—he saw these as causal steps in the same process (Gayon 2016; Lankester 1890a). In this way, Weismann positioned random mating (panmixia) as both the natural state of a population in the absence of selection and a causal mechanism which would act to reverse the effects of selection on a trait (Lankester 1890b; Rose et al. 2008; Weismann 1891).

## 3 Panmixia: From Reversing Selection to Model Assumption

A major motive behind Weismann's description of panmixia was to excise Lamarckian explanations from evolutionary theory and argue that only Darwinian natural selection was necessary as a mechanism to prevent reversion. In the following decades, the problem of reversion would recede somewhat to the periphery of evolutionary discussions. The concept of panmixia, nevertheless, grew in influence largely through the attendant assumption that free intercrossing or random breeding represented something akin to the ‘natural state’ of a population. In the years around 1900, this biological assumption began to be routinely incorporated into biometry, the study of heredity using statistics.

In his work *Mathematical Contributions to the Theory of Evolution III*, Karl Pearson defines the concept of panmixia mathematically (Pearson 1896). This led Pearson to observe that ‘the difficulty is not the establishment of panmixia, but as to what is to be considered the “original general population”… how is it possible to pick out any particular stage of general population as the “focus of regression”’ (Pearson 1896). In 1898, Pearson goes further, citing his expansion of Galton's ‘Law of Ancestral Heredity’ in which he abandons the idea of panmixia as a cause of reversion, and states ‘there is no workable theory of heredity yet discovered which favours in any way degeneration by panmixia’ (Galton 1889; Pearson 1898).

Here, Pearson is specifically referring to panmixia's role in causing reversion. In an 1896 paper, he identified that both Weismann's panmixia and Galton's theory of regression relied on the hypothesis of ‘free mating’ in the absence of selection (Gayon 2016). In the same paper, he developed his theory of Ancestral Heredity and continued to apply this assumption to demonstrate how under relaxation of selection, no regression would occur and ‘stability’ would persist (Pearson 1896). Pearson retained this view until the end of his career. In one of his last works, he explicitly outlines the role he believed panmixia plays in maintaining this stability. He wrote that ‘[t]he force which preserves the type in any species is summed up in the words random mating, or the whole deviations in excess and defect of type interbreed and cancel out, thus perpetuating mediocrity, and keeping the type stable*. Stability generally, as on the Mendelian hypothesis, depends on random mating…​​ *“Panmixia” does not signify, as Weismann held, regression, but stability of type’ (Pearson 1930). Although Pearson disagreed with Weismann that panmixia would lead to reversion, he is arguing that it is not merely a mathematical simplification, but a biologically significant process necessary for maintaining stability in a population.

Pearson's theory of Ancestral Heredity was built on ideas from Galton's (1889) book *Natural Inheritance*. In this, Galton introduces a method of determining heritability using regression analyses. This method rested on Galton's earlier Law of Physiology where crucially ‘the person may be accepted on the whole as a fair representative of the germ’ (Galton 1897) and the contributions of the ancestor to the germ plasm are greater if they are more closely related. Galton suggested in his law of Ancestral Heredity that each successive generation contributed one half, a quarter, one eighth and so forth of their germ plasm to an individual.

To demonstrate this, he relied on the assumption that the only cause of correlation between traits was heredity, where pairings between individuals are random with respect to the trait (i.e. random mating). Galton's views changed over time about whether this assumption was true in human populations. In 1869, he stated that intellectual people had ‘a tendency [to mate] “like to like”’ (Galton 1869, 325). However, after devoting significant work to assessing the strength of this assumption and other forms of assortative mating (Galton 1889, Chapter 7), he wrote in 1889 that ‘I find little indication in the average results obtained from a fairly large number of cases, of any single measurable personal peculiarity, whether it be stature, temper, eye‐colour, or artistic tastes, in influencing marriage selection to a notable degree’ (Galton 1889, 85). This view appears to have been short lived, as in 1909 he once again wrote that ‘[on the subject of] Marriage of like to like—In each class of society there is a strong tendency to intermarriage’ (Galton 1909, 19).

Galton explained why fingerprints proved so useful in his studies: ‘finger patterns are apparently the only peculiarity in which Panmixia, or the effect of promiscuous marriages, admits of being studied on a large scale’… ‘The result of Panmixia in finger markings, corroborates the arguments I have used in Natural Inheritance and elsewhere, to show that “organic stability” is the primary factor by which the distinctions between genera are maintained’ (Galton 1892, 20). Here, Galton's initial goal appeared to be finding a trait for which mating is effectively random, whilst maintaining that this was rare. Despite him considering that panmixia was likely to be rare in humans, it became a central assumption in Galton's analyses, largely because it facilitated his regression method.

This approach continued when these methods were later built upon by Pearson, who states that under random mating ‘all characters will be inherited at the same rate’ (Pearson 1900). Therefore, Galton's theory that ‘the mean character of the offspring could be calculated… [from] knowledge of the corresponding characters in ancestry’ (Yule 1902) became a core principle in the work of the emerging biometrical movement (Provine 2001). Pearson expressed more confidence than Galton, stating that ‘[m]en and women do not mate at random; our measurements and observations show that for practically all characters there is a selective mating’ (Pearson 1912, 36). However, he continued to apply the assumption of panmixia in order to uncover the principles of heredity.

This frequent application of panmixia in Galton and Pearson's work, besides its mathematical convenience, may also have been because both men appeared to interpret it differently than we do today. Although in principle panmixia assumes total random mating with respect to geography, relatedness, phenotype, genotype and sex ratios, both Galton and Pearson appear to consider panmixia in a much narrower sense. Specifically, they seemed to argue that a population would mate randomly with respect to a trait if preferential mating for that trait was absent. For example, when Galton argues why people mate panmictically with respect to their fingerprints, he says that ‘it would be absurd… to assert that in the struggle for existence, a person with, say, a loop on his right middle finger has… a better chance of early marriage’ (Galton 1892, 19). Pearson is even more explicit, stating: ‘[as] opposed to all these forms of selective mating, we have:‐… Pangamic mating, or the mating at random of all members within the race’ (Pearson 1900, 484) (Note: here Pearson defines ‘pangamic’ in the same way he later refers to panmixia [Pearson 1930]).

Interestingly, both Galton and Pearson do occasionally mention barriers to panmixia other than preferential mating: ‘[intelligent women] would be likely not to marry so much or so soon as other women, because…their society were restricted to the persons in their immediate neighbourhood’ (Galton 1869, 328) and ‘religious society is particularly large… therefore the necessity of choosing a pious husband is no material hindrance to the marriage of a near female relation of an eminent divine’ (Galton 1869, 329). These hint at him thinking about the non‐randomness of mating due to constrained geography or mate choice, albeit as passing mentions within his voluminous writing on heredity.

Pearson too considered, though more explicitly rejected, the role of geography stating: ‘another explanation of these high coefficients of assortative mating has been suggested to me, namely that the population of England is built up of a number of local races, and that men and women mate within their locality. Now it appears to me that this argument would be far more valid, if my material was drawn in bulk from local lower middle and artisan classes. But it is very doubtful how far it is true of the middle classes, such as provide the students at the London colleges. The middle classes undoubtedly marry in their own “sets,” but these are hardly local sets’ (Pearson and Lee 1903). The fact that both men were specifically interested in determining the heritability of traits within the strata of Victorian society was perhaps the reason they neglected the non‐randomness with mating with respect to geography. If your study group consists of ‘men of eminence’, such as fellows of Oxford and Cambridge, or English judges, as Galton's often did, these insular groups would likely exhibit very little significant effects of isolation by distance.

In the second edition of his influential and popular science book, *The Grammar of Science*, Pearson goes further, linking but decoupling the concept of panmixia to speciation: ‘[i]f all members of a race are equally fertile and they continue to pair at random, then a permanent differentiation into mutually infertile sections,‐ an origin of species,—seems impossible’ (Pearson 1900, 421). He continues that ‘[w]ithout such selection, however, neither self‐fertilisation nor mating of like with like necessarily connotes a change of type. Natural selection requires selective mating, sexual selec­tion in its broadest sense, to produce that barrier to inter­ crossing on which the origin of species depends… This [sexual selection] I should prefer to term preferential mating’ (Pearson 1900, 423). He concludes that ‘[i]f natural selection be at work, all the forms [of preferential mating] can have great influence on differentiation—it is only [random mating] which would check it’. In a stable system (in other words, where no differentiation or evolution is taking place), he argues panmixia would be the norm. Later in the book, he states this explicitly ‘[l]et us suppose that mating is pangamic, that the race is sensibly stable’ (Pearson 1900, 479). Thus, Pearson sees panmixia not only as a mathematical simplification but also as a biologically consequential process. He appears to argue that panmixia is the null state of a population, in the absence of selection or other evolutionary forces. This a similar position to that held by Weissman, but without the inclusion of panmixia leading to reversion.

## 4 The Panmictic Population and Mendelism

By 1900, the works of Gregor Mendel, which revealed the statistical rules by which monogenic traits are inherited, had been translated into English and published. In the years that followed, there was increasing interest in reconciling Mendel's rules with the work of the biometricians (Lock 1906, Chapter 8; Pence 2022, Chapter 5; Thomson 1908; Yule 1902, Chapter 10). One of the first examples of this was in 1902 when Pearson's former student, Udny Yule (Froggatt and Nevin 1971; Provine 2001), took Pearson's law of Ancestral Heredity (see Box 1) and applied Mendelian principles to it to determine if certain Mendelian principles might lead to a more general biometric law (Tabery 2004). To that end, he states ‘the first question to be asked… [is] what, exactly, happens if the two [phenotypes] are left to themselves to inter‐cross freely as if they were one race’ (Yule 1902). He discovered that, assuming complete dominance, when offspring of the hybrid generation bred at random, it produced a stable 3:1 equilibrium and declared that ‘Mendel's laws, so far from being in any way inconsistent with the Law of Ancestral Heredity, lead directly to a special case of that law’ (Yule 1902). Through this work, Yule can be credited with introducing the assumption of random mating into Mendelian population genetics (Edwards 2008).

**BOX 1 The meanings of several key terms in this paper have changed substantially over time. The below serves as an indication of what they would have meant both to major figures and at different periods**

**Table:** 

Changing meanings	
Panmixia	Mid‐19th century: A biological process in the absence of selection, leading to random mating and resultant ‘reversion’ to an ancestral state. Late 19th century–Modern Synthesis: The assumption of random mating, usually with respect to phenotype. After the Modern Synthesis: The assumption of total random mating with respect to geography, relatedness, phenotype, genotype and sex ratios
Heredity	Mid‐19th century: Revolving around Darwin's theory of ‘pangenesis’, ‘the first comprehensive theory of heredity’ (Moore 1972, 8)—this model of heredity suggested that ‘gemmules’ were shed by parental cells then transmitted into offspring via the gonads, so that qualities of the parents are blended (Darwin 1868, Chapter 27). This was essentially a Lamarkian idea and can be traced back to Hippocrates's ideas over 2000 years earlier (Zirkle 1946). To Galton: A natural law that states that all traits are inherited ‘in a marked and equal degree’. He focused on ‘biparental inheritance, reversion to ancestral characters, and the rejection of any significant role for the inheritance of acquired characters’ (Bulmer 1999). Early 20th century: Integration of Mendelian inheritance, and recognition that ‘Heredity is no entity, no force, no principle, but a convenient term for the genetic relation between successive generations, and inheritance includes all that the organism is or has to start with in virtue of its hereditary relation’ (Thomson 1908, 6).
Law of Ancestral Heredity/Law of Physiology	Galton believed in blending inheritance and thought that heritable variation was continuous and normally distributed (Magnello 1998). Thus, his law of Ancestral Heredity stipulated that an individual's relationship to their ancestors should follow a simple geometric pattern (one half to parents, a quarter to grandparents etc.). To Pearson, ‘A purely statistical (as distinguished from a mechanical or physiological) theory of heredity [that] may be used to express Galton's Law of Ancestral Heredity’ or simply ‘the mathematical expression of statistical variates’ (Magnello 1998).
Reversion/regression/regression analyses	Early 19th century: An evolutionary force where traits revert to ancestral states under certain circumstances (such as domestication). Mid‐19th century: Galton develops his regression analyses to study the above biological process. Late 19th century–Modern: Belief in biological regression to ancestral states diminishes. Regression analyses continue to be used to study correlation between variables, but its results are viewed as resulting from statistical effects rather than biological ones.

Two years later, Pearson also attempted to reconcile Mendel's laws with his law of Ancestral Heredity. He set out to discover whether ‘(a) after hybridisation, do or do not the offspring of the hybrids, if mated at random inter se, give a stable population? (b) If they do give a stable population, do the ancestral correlations diminish or not in a geometrical series?’ and that ‘if the offspring of hybrids mated at random give a ‘stable’ population, then we ought to be able to predict at least certain phenomena with regard to the result of crossing its constituents’ (Barrington et al. 1904).

Similarly, when Pearson addresses these questions mathematically and develops the ideas presented in Yule (1902), he states that ‘we suppose… the members of the second generation to cross absolutely at random and with equal fertility*’ adding that ‘*If one is to study heredity in populations with a view to the problem of evolution, the conditions as to fertilisation should approach as far as possible the conditions we suppose them to be under in a natural state; we must fix our attention on the mass relations between successive generations of the population’ (Pearson 1904). Here, Pearson is arguing that, whatever the actualities of human mating, we can in general take the natural state of a ‘stable’ population to be ‘sensibly mating at random’ (Pearson 1909).

## 5 The Panmictic Population as a Dominant Model

Despite quashing panmixia as a mechanism for reversion, Pearson—sceptical of Mendelism—contributed significantly to the entrenchment of random mating as a core assumption about the new idealised Mendelian populations through his mathematical work of the early 1900s. Panmixia's presence only increased in subsequent years as researchers continued to explore the statistical implications of the Mendelian theory of inheritance.

To model the effects of migration, selection and other dynamics on allele frequencies, the first population geneticists needed a reasonable null model of a population on which to study these. They moved from Galton and Pearson's conceptualisations of populations as statistical groupings to idealised models of natural systems (Hey 2011), retaining Pearson and Yule's assumption that they are panmictic (Fisher 1918, 1922a, 1930; Hardy 1908) at least in part to simplify the mathematics they were developing (Haldane 1964). This allowed for allele frequencies to be treated as probabilities and for these to be drawn from a chosen distribution (Fisher 1922b).

Perhaps the most famous population genetic model was developed independently by Godfrey Hardy (1908) and Wilhelm Weinberg (1908), and it became known as the Hardy–Weinberg Principle. They showed that the allele frequencies in the next generation of a population could be predicted from genotype frequencies in the current one, if they followed a number of assumptions (panmixia, infinite size, isolation, dominance status and using traits with single bi‐allelic autosomal locus). For instance, under panmixia, the probability of two genotypes mating in a population is simply the product of their frequencies. This result also requires the assumption of an infinitely large population, as there is no risk the supply of potential mates will run out, or indeed genetic drift. Thus, the selection of both mates out of the population can be independent, allowing their probabilities to be treated as equivalent and multiplied to calculate a joint probability.

Notably, panmixia was not incorporated as a de novo assumption during the development of Hardy's model. Hardy stated that he carried out the work in response to the geneticist Reginald Punnett's question of what would happen to genotype frequencies under random mating (Edwards 2008). Punnett's question was directly influenced by Yule's (1902) modelling of Mendelian Inheritance under random mating. Yule had criticised a lecture Punnett gave earlier that year, stating that ‘one could only form some theory on the assumption of random mating’, and argued that under panmixia, Mendelian inheritance would result in a runaway increase in trait frequencies (Punnett 1908). We speculate that this was probably due to a misunderstanding on his (and Pearson's part) ‘that implicit in the Mendelian theory was the assumption that the gene frequency was one‐half, for then indeed a 3:1 ratio would appear and be maintained’ (Edwards 2008). In other words, Yule appeared to think that Mendel argued all gene frequencies were one half, so his law stipulated that in the next generation, three quarters of the offspring would have at least one dominant allele, ultimately leading to the fixation of that allele.

Punnett asked Hardy if, under Yule's assumption of random mating, this was really the case. Punnett ‘put [his] problem to him as a mathematical one’ (Punnett 1950). Hardy was not intending to represent that natural world in his model, stating ‘I have, of course, considered only the very simplest hypotheses possible. Hypotheses other than that of purely random mating will give different results’. Hardy was developing mathematics for a model with pre‐existing assumptions given to him, the Biometricians’ model, and implies that if he had been given different assumptions to work with, he would have produced a different model. The subsequent dominance of the Hardy–Weinberg model as the starting point for both teaching population genetics and building more complex population genetic models ensured that the idea that panmixia ‘represented the ideal state of a sexually reproducing population unaffected by any evolutionary force’ would be effectively baked into the foundations of the field (Gayon 1998, 298).

This concept was further cemented a few years later through the work of Ronald Fisher who, specifically building on the contributions of Pearson and Yule, developed the first mathematical models of how Mendel's laws of inheritance would affect whole populations (Fisher 1918). Fisher, despite citing neither Hardy or Weinberg, continued the use of single, infinitely large panmictic populations, which allowed him to infer the probability distributions of allele frequencies within them and effectively ignore the effect of stochastic factors on these frequencies (Fisher 1922a; Haldane 1964; Majumder 1992; Provine 1986). Through this approach, Fisher argued the primary driver of evolution must be deterministic selection acting on single genes. At the same time, Fisher's simplifying assumptions allowed him to develop many of the principles and methods which laid the groundwork for modern population genetics, and are still validly used in the field today (Charlesworth and Charlesworth 2017; Crow 1987).

## 6 Challenging the Panmictic Population

One of the aims of this paper is to initiate a conversation about the continuing usefulness of proceeding from a starting assumption of randomly mating populations. As we discuss in the next section, we believe this is a timely concern. However, it is not a new one. The panmictic population concept has faced criticism since close to its inception, much of it focused on the idea's biological implausibility.

Fisher's work was scrutinised on this front. In reviewing Fisher's seminal 1918 paper, Punnett admits that ‘I have had another go at this paper but frankly I do not follow it owing to my ignorance of mathematics’. Nevertheless, he questions the reasonableness of Fisher's assumptions: ‘I do not feel that this kind of work affects us biologists much at present. It is too much of the order of problem that deals with weightless elephants upon frictionless surfaces, where at the same time we are largely ignorant of the other properties of the said elephants and surfaces’ (Norton et al. 1976).

The American geneticist Sewall Wright was another significant critic who found Fisher's assumptions—for example the effective size of a population being the ‘total population of the planet’ (Fisher 1929)—to be biologically unrealistic (Mayr 1982; Provine 1986). Although many of Wright's objections to large panmictic populations stemmed from his view that small partially isolated populations gave the most favourable conditions for evolution, his objections included the unrealistic nature of panmixia: ‘random breeding [is] a condition not realized in natural species as wholes. In most cases random interbreeding is more or less restricted to small localities’ (Wright 1929). These misgivings led Wright to formulate theories that moved away from the panmictic population concept and towards other more biologically realistic models such as effective population size (Wright 1931), the island model (Wright 1931) or isolation by distance (Wright 1943).

Fisher himself later acknowledged that panmixia was a ‘simplifying, but unrealistic, assumption’ (Fisher 1941). Indeed, he developed models of assortative mating (Fisher 1922a) and the spread of an advantageous allele in (one dimensional) continuous space (Fisher 1937), neither of which assumes panmixia. Nevertheless, it has been argued that ‘[Fisher] clearly believed that an entire species for the purpose of statistical analysis of evolution was a random breeding Mendelian population’ (Provine 1986, 255). Fisher's commitment to the assumption of infinitely large panmictic populations both stemmed from and supported his conviction that mass selection on quasi‐independent loci is the primary motor of adaptive evolutionary change. Wright, meanwhile, in building models to more closely reflect the structure of natural populations as finite and subdivided, gave a much greater evolutionary role to stochastic processes, such as genetic drift. The debate between Fisher and Wright, and latterly their followers, over both the primary causes of genetic evolution and the best approaches to modelling them, fundamentally shaped the developing field of population genetics through the rest of the 20th century and beyond (Skipper 2002, 2009). The works of both remain fundamental and profoundly influential on population genetic modelling today.

In 1959, the evolutionary biologist Ernst Mayr launched a spirited critique of the contributions of the early theoretical population genetics to the unfolding synthesis (Rao and Nanjundiah 2011). Where most peers considered the seminal books and papers of Fisher, Wright and Haldane as laying the groundwork of neo‐Darwinian theory, Mayr dismissed them as biologically uninformed and misguided in their elevation of the ‘gene pool’ over a traditional organismal perspective and their relative neglect of core evolutionary issues such as speciation. As an aside, the entrenchment of the panmictic population concept has been credited as introducing the concept of a gene pool (Gayon 1998, 298).

Responding to Mayr's criticisms on what he called ‘beanbag genetics’, Haldane conceded that much of the work in early theoretical population genetics, even Wright's more biologically realistic models, was ‘no doubt … too simple’ (Haldane 1964). Nevertheless, Haldane defended the usefulness of modelling in evolutionary theory in general and held that mathematical works on these problems, even those later rendered outmoded or superfluous, had been more than worth the effort. ‘[T]he existing theories of population genetics,’ he wrote, ‘will no doubt be simplified and systematised. Many of them will have no more final importance than a good deal of nineteenth‐century dynamical theory. This does not mean that they have been a useless exercise of algebraical ingenuity. One must try many possibilities before one reaches even partial truth’ (Haldane 1964).

As we have seen, the positioning of random mating as the default state in natural populations was entrenched before the Modern Synthesis. It lived on in the work of the founders of population genetics, and especially that of Fisher, due to both its mathematical and computational convenience and the influence of certain biological assumptions about the nature of populations and the main forces driving evolution. In Haldane's view, the early population geneticists had done the best they could with both the scant genetic data and the limited computational resources at their disposal. In this spirit, we can both applaud the foundational work done in population genetic modelling using the null assumption of randomly breeding populations, while recognising the need to revisit and where possible replace this with assumptions that more accurately reflect our current knowledge of the biology of natural populations.

## 7 The Panmictic Population in the Genomics Era

The majority of statistical approaches for inferring population history in evolutionary genetics today assume panmixia within one or more populations. These include the hugely influential programs STRUCTURE (Pritchard et al. 2000) and ADMIXTURE (Alexander et al. 2009) both of which infer source population structure and ancestry proportions for sampled genomic data. Similarly, the suite of tools referred to as *F*‐statistics (Patterson et al. 2012; Reich et al. 2009), and their derivatives qpADM, qpGraph and qpWave (Haak et al. 2015; Patterson et al. 2012), assume panmictic subpopulations, albeit with admixture. Tools used to infer past effective population size change, such as PSMC (Li and Durbin 2011) and MSMC (Schiffels and Durbin 2014), while not necessarily based on the assumption of panmixia, require that assumption if their outputs are to be interpreted as reflecting real population size changes. Even more recent approaches which aim to fully model linkage disequilibrium, such as fineSTRUCTURE (Lawson et al. 2012) and MOSAIC (Salter‐Townshend and Myers 2019), still assume panmixia within source populations. Although structured coalescent models have been developed, these and the majority of coalescent‐based methods model two or more panmictic populations connected by gene flow (Hey 1991; Kingman 1982; Wakeley 2009, Chapter 3).

The approaches mentioned above, and others, have been spectacularly useful, leading to seminal discoveries including Neanderthal admixture (e.g. Green et al. 2010), a plethora of past migratory processes (e.g. Bramanti et al. 2009; Haak et al. 2015; Malaspinas et al. 2016; Rasmussen et al. 2011; Reich et al. 2012; Skoglund et al. 2012) and inferences on past population size change (e.g. Li and Durbin 2011; Mallick et al. 2016). Furthermore, the developers of these approaches are very clear about the assumptions made and often explicitly point out that their models do not perfectly replicate biological reality (e.g. Pritchard et al. 2000) and have only been able to make these path‐dependent advances by building upon a century of theoretical innovation developed on the foundations of the panmictic population model.

It could be argued though that genetic data are never sampled from one or any number of panmictic (sub‐)populations and that the appropriate null model for genetic variation in space is isolation‐by‐distance (Wright 1943). Indeed, a number of studies indicate that assuming panmixia can lead to erroneous conclusions (e.g. Eriksson and Manica 2012; Frantz et al. 2009; Mazet et al. 2016; Städler et al. 2009; Tournebize and Chikhi 2025; Wakeley 1999). Several techniques have been developed to study evolution from genetic data by assuming or approximating spatial continuity. However, there are considerable challenges in developing the mathematical tools needed to model genetic variation in continuous space. A step towards true continuous space models is demic models, whereby space is discretised into a grid of demes or cells—each modelled as a panmictic sub‐population. The assumption here is that as grid resolution (i.e. the number of demes) increases, the overall model tends towards a continuous space model (Barbujani et al. 1995; Currat et al. 2004; Itan et al. 2009; Racimo et al. 2020; Ray et al. 2003). A small number of inferential approaches have also been developed which model genetic variation as continuous across space (e.g. Loog et al. 2017; Miller 2005; Schmid and Schiffels 2023). These currently have limited utility and are by no means the predominant inferential approaches in the field (Battey et al. 2020; Bradburd and Ralph 2019).

## 8 Discussion

In the introduction to Wright's *Evolution in Mendelian Populations* he wrote that ‘species [are] an intricate network of living matter, physically continuous in space‐time’ (Wright 1931). Given it would be over 70 years before the analysis of human genome data would demonstrate this at a molecular level (Prugnolle et al. 2005; Serre and Pääbo 2004), it was a remarkably prescient observation.

The adoption and continued use of panmixia in the Modern Synthesis was not merely a mathematical convenience but also the result of the concept being already embedded in the field. The panmictic population concept has its roots in the idea that organisms are descended from distinct ancestral ‘pure’ populations which mate at random and that panmixia is the mechanism for reversion to this ‘null’ state. It was further entrenched by Pearson's belief that random mating kept natural populations ‘stable’ and was a necessary component of his (and Galton's) Biometrical analyses. By the time of the Modern Synthesis, although reversion and Galton's regression approach were no longer prominent in the field, panmixia had become the default assumption when building population genetic models. Though many modern practitioners might question the assumption of panmixia, it has persisted in practice due in large part to its mathematical and computational convenience. This is not to deny its enormous and ongoing value in the field and its role in many important discoveries.

It is tempting to imagine what the field of population genetics would look like today if the assumption of panmixia had not become embedded within it by the time of the Modern Synthesis. Fisher's work on the spread of selected alleles in (one dimensional) space (Fisher 1937) and Wright's development of the Isolation by Distance (Wright 1943) model provide clear evidence that the attempts were made early on to think outside the panmictic population concept. Had these gained more traction, and had Wright's work superseded Fisher's in terms of influence, it is conceivable that over the last 100 years, population genetics may have been built on different foundations.

Perhaps the major reason continuous space models remain in their relative infancy is that, unlike those based on the panmictic population concept, they have not benefited from a century of theoretical development as the central paradigm in evolutionary genetics. Today, many population geneticists are beginning to regard the panmictic population as a limiting assumption as opposed to a simplifying one. As interest in alternative approaches grows, now is the ideal time to evaluate its continued use and develop operationally simpler tools based on alternative, more biologically realistic models. With the increase in large‐scale genomic databases and cheap computational power, there has never been a better moment for researchers to examine the assumptions embedded in their tools and to consider whether they are still necessary today.

The panmixia assumption places limits on population genetics. It may also help keep alive certain outmoded ways of thinking about human populations and human variation. The theoretical foundations of population genetics were laid down by individuals who subscribed to a very different picture of human variation, one which grouped our species into several discrete biological races separated by fundamental and innate differences (MacKenzie 1981; Rutherford 2021). While these views are widely rejected by contemporary population geneticists, human genomic diversity studies often target populations which are assumed not to have undergone recent ‘admixture’ (Lipphardt 2012; Reardon 2005, Chapter 3; Widmer 2014), potentially misrepresenting patterns of genetic diversity in space and perpetuating notions of discrete panmictic ancestral populations.

It is important to stress that we do not see embedding of the panmictic population model as an epistemological ‘frozen accident’. Because of its demonstrable utility, it was perhaps inevitable that panmixia played a major role in the development of population genetic theory. Rather, we advocate for recognition of its history and limitations and for the development of additional approaches that recognise the continuous nature of spatial population structure.

## Author Contributions

Andy Walton is the primary author; all others contributed equally to the writing and editing.

## Conflicts of Interest

The authors declare no conflicts of interest.

## Data Availability

Data sharing not applicable to this article as no datasets were generated or analysed during the current study.
